# Interference Expression of *StMSD* Inhibited the Deposition of Suberin and Lignin at Wounds of Potato Tubers by Reducing the Production of H_2_O_2_

**DOI:** 10.3390/antiox11101901

**Published:** 2022-09-25

**Authors:** Ying-Yue Ren, Hong Jiang, Li Ma, Jiang-Wei Yang, Huai-Jun Si, Jiang-Ping Bai, Dov Prusky, Yang Bi

**Affiliations:** 1 College of Food Science and Engineering, Gansu Agricultural University, Lanzhou 730070, China; 2 College of Life Science and Technology, Gansu Agricultural University, Lanzhou 730070, China; 3 College of Agronomy, Gansu Agricultural University, Lanzhou 730070, China; 4 Department of Postharvest Science, Agricultural Research Organization, Rishon LeZion 7505101, Israel

**Keywords:** *Solanum* *tuberosum* L., *StMSD*, interference expression, wound healing, H_2_O_2_, phenylpropanoid metabolism

## Abstract

Superoxide dismutase (SOD) actively participates in the wound stress of plants. However, whether *StMSD* mediates the generation of H_2_O_2_ and the deposition of suberin polyphenolic and lignin at potato tuber wounds is elusive. In this study, we developed the *StMSD* interference expression of potato plants and tubers by *Agrobacterium tumefaciens*-mediated transformation. The *StSOD* expression showed a marked downregulation in *StMSD*-interference tubers, especially *StCSD2* and *StCSD3*. The content of O_2_^•^^−^ exhibited a noticeable increase together with the inhibition in H_2_O_2_ accumulation. Moreover, the gene expression levels of *StPAL* (*phenylalanine ammonia-lyase*) and *StC4H* (*cinnamate-4-hydroxylase*) were downregulated in *StMSD*-interference tubers, and less suberin polyphenolic and lignin depositions at the wounds were observed. Taken together, the interference expression of *StMSD* can result in less suberin polyphenolic and lignin deposition by inhibiting the disproportionation of O_2_^•−^ to H_2_O_2_ and restraining phenylpropanoid metabolism in tubers.

## 1. Introduction 

Reactive oxygen species (ROS) is considered as playing a central role in the wound healing of potato tubers [1]. They serve as a priming signal that activates the defense responses contributing to the substrate synthesis of healing tissues [2] and are also used directly for the oxidative crosslinking of the substrate [3]. The generation of ROSs required for the healing of tubers mainly comes from NADPH oxidase (NOX), which generates O_2_^•−^ by transferring NADPH electrons to O_2_. However, O_2_^•−^ is unstable and is quickly converted into the more stable H_2_O_2_ via the action of superoxide dismutase (SOD) [4,5].

SOD family members are divided into three types, namely Cu/Zn-SOD (CSD), iron SOD (FSD), and manganese SOD (MSD), according to their different metal cofactors in plants [6]. Each type of SOD has a disproportionation function that catalyzes O_2_^•−^ to H_2_O_2_ [7]. Previous studies have revealed that SODs play an active role in plant response to wound stress [8]. For example, CSD expression was induced by wound stress in capsicum and tomato leaves, thereby promoting the accumulation of H_2_O_2_ [9]. The upregulation of MSD expression caused by wound stress was required for excess H_2_O_2_ production in carrots [10]. Our recent research found that wound stress led to different degrees of upregulated expression of *StSODs* in potato tubers, thus resulting in H_2_O_2_ generation [6]. Additionally, studies have shown that H_2_O_2_ generated from O_2_^•−^ disproportioned by SOD is associated with the activation of phenylpropanoid metabolism as signal molecules. For example, wound stress upregulated the *DcMSD* expression and improved the H_2_O_2_ content, leading to induced expression of *PAL*, *C4H,* and *4CL* with concomitant accumulation of phenolics in carrots [10]. An increased H_2_O_2_ content caused by overexpression of *PaSOD* from Himalaya cinquefoil in potato plants improved the transcription of *PAL*, *C4H*, and *4CL*, which promoted lignin accumulation [11]. In addition, SOD directly participates in the oxidative cross-linking of cell wall components as an oxidant by yielding H_2_O_2_. A CSD in Scots pine was involved in the lignification process by supplying H_2_O_2_ in wounding responses [12]. The generation of H_2_O_2_ via the disproportionation of apoplastic CSD was correlated with the formation of suberin and lignin in spinach hypocotyls [13]. 

While it has been reported that wound stress could upregulate *MSD* expression, promote H_2_O_2_ accumulation, and regulate phenylpropanoid metabolism, little information is known about the molecular function verification of the *StMSD* gene in regulating phenylpropanoid metabolism by yielding H_2_O_2_ and affecting suberin polyphenolic and lignin deposition in potato tubers during healing. In this paper, *StMSD* (Soltu.DM.06G011380) was selected for gene cloning and interference-expression vector construction. The *StMSD* interference-expression potato plants and tubers were generated by *Agrobacterium tumefaciens* transformation. The role of *StMSD* involved in healing was investigated by determining the transcription levels of *StSODs*, *StPAL* and *StC4H*, together with the assay of O_2_^•−^ and H_2_O_2_ content, and the visualization of suberin polyphenolic and lignin deposition at *StMSD* interference expression of tuber wounds during healing.

## 2. Materials and Methods

### 2.1. Potato Plantlets

Potato (*Solanum tuberosum* L. cv. Atlantic) plantlets were obtained from the Molecular Biology Laboratory in the College of Life Science and Technology, Gansu Agricultural University.

### 2.2. Growth Conditions of Potato Plantlets

The wild-type and interference expression of potato plantlets were grown on Murashige and Skoog (MS) medium with 3% sucrose by sub-culturing under constant conditions for 8 h in the dark and 16 h under light with a lamp of 20,000 lx at 23 ± 2 °C. After culturing for a month, 4- to 5-week-old plantlets were transferred to another MS medium with 8% sucrose for about 60 d and then placed in the dark for 30 d with the purpose of the induction of microtubers. 

### 2.3. Creation of StMSD Interference Expression of Potato Plants and Tubers

The partial gene fragment (327 bp) was amplified using the primers designed from the coding region of *StMSD* gene (*StMSD*-F: 5′-GGGGACAAGTTTGTACAAAAAAGCAGGCT-3′; *StMSD*-R: 5′-GGGGACCACTTTGTACAAGAAAGCTGGGT-3′) and cloned into plasmid pHellsgate8 by gateway cloning technology, then named as an interference expression (pHellsgate8-*StMSD*), which was then delivered into *Agrobacterium* strain (LBA4404), as previously described [14]. 

Potato transformation was carried out following the method described previously [15]. After removing the bud eye totally, the microtubers collected from Section 2.2 were cut into 2–3 mm thick slices and immersed in a solution of *A. tumefaciens* containing pHellsgate8-*StMSD* plasmid and empty vector for 8 min. The infected slices were co-cultured at 28 °C for 48 h in the dark on MS medium with 3% sucrose, as mentioned in Section 2.2, transferred into differentiation medium (zeatin (ZT, 2 mg L^−1^), indole-3-acetic acid (IAA, 1 mg L^−1^), gibberellin (GA, 0.5 mg L^−1^), 6-Benzylaminopurine (6-BA, 0.5 mg L^−1^), MS with 3% sucrose, kanamycin (75 mg L^−1^), and carbenicillin (200 mg L^−1^)), and cultured for 8 h in the dark and 16 h under light with a lamp of 20,000 lx at 23 °C. The new buds derived from the slices were cut and inserted into the rooting MS medium containing kanamycin to screen kanamycin-resistant transformed plantlets. After 1–2 months, some of the buds that could come into root were considered to be kanamycin-resistant transformed plantlets and then grown in flasks.

Genomic DNA was extracted from *StMSD* interference-expression and wild-type plantlets according to the genomic DNA extraction kit instruction (Cat. No. DP305, TianGen Biotech, China) and was used to perform polymerase chain reaction (PCR) with the primers of neomycin phosphate transferase (*NPT II*) gene (F: GCTATGACTGGACAACACAG; R: ATACCGTAAAG CACGAGAA) to screen kanamycin-resistant potato plantlets. The PCR amplification reaction included 1 μL of Phanta Max Super-Fidelity DNA Polymerase, 2 μL of upstream and downstream primers, 1 μL of cDNA, 1 μL of dNTP Mix, 25 μL of 2 × Phenta Max Buffer, and 18 μL of ddH_2_O. The PCR cycling conditions were set as follows: 95 °C for 10 min and 30 cycles of 95 °C for 10 s, 58 °C for 20 s, and 72 °C for 30 s. The pHellsgate 8-*StMSD* plasmid served as the positive control. The positive plantlets were obtained for the induction of microtuber. 

### 2.4. Wounding and Wound Healing of Tubers

Referring to a previous method [16], *StMSD* interference-expression tubers collected from the four selected interference-expression lines were used for the assessment, and wild-type tubers were selected randomly, and cut in half with the sterilized knife, then placed into perforated polythene bags (15 cm × 27 cm, thickness 0.02 mm) at constant temperature and relative humidity conditions (20–25 °C, RH 80–90%) in the dark. 

### 2.5. Sampling

Healing tissues were obtained from a 2 mm thickness below the wounds at 0 d, 1 d, 3 d, 5 d, and 7 d; ground into powder in liquid nitrogen; and collected into centrifuge tube, as described previously [16]. The IntLn 1 line with the most marked interference expression was chosen for observation of the autofluorescence of suberin polyphenolic and lignin.

#### Gene Expression of StSODs, StPAL, and StC4H

The total RNA was extracted from the wild-type and interference-expression tubers using the RNA extraction kit (Cat. No. DP419, TianGen Biotech, Beijing, China), and the first-strand cDNA was reverse transcribed using a TIAN script RT kit (Cat. No. KR116, TianGen Biotech, China) for real-time fluorescence quantification PCR. The cDNA was used for assaying the *StSODs*, *StPAL*, and *StC4H* by quantitative PCR reaction with SYBR Green PCR kit (Cat. No. FP205, TianGen Biotech, China). The PCR amplification reaction contained 10 μL of 2 × SuperReal PreMix Plus, 0.6 μL of upstream and downstream primers, 1.5 μL of cDNA, 0.4 μL of 50 × ROX Reference Dye, and 6.9 μL of ddH_2_O. The PCR cycling conditions were set as follows: 95 °C for 10 min and 45 cycles of 95 °C for 10 s, 55 °C for 20 s, and 72 °C for 30 s. The specific primers required for the experiments are shown in the Supplementary Material (Table S1). The calculation of expression level was carried out based on the 2 ^(−ΔΔ CT)^ method [17]. 

### 2.6. O_2_^•^^−^ and H_2_O_2_ Content

The superoxide anion (O_2_^•−^) content was assayed according to the kit instruction (Shanghai Sinobest Biotech, Shanghai, China). Frozen samples were mixed with 5 mL of extraction reagent, centrifuged at 10,000× *g* for 10 min, and the supernatant was collected for further assay. The absorbance was recorded at 530 nm (O_2_^•−^ oxidizes hydroxylamine to produce a red azo compound which has a characteristic absorption peak at this wavelength) and used for the calculation of O_2_^•−^ content, which was expressed as mmol kg^−1^ on the basis of fresh weight.

The hydrogen peroxide (H_2_O_2_) content was detected in light of the kit instruction (Nanjing Jiancheng Biotech, Nanjing, China). Frozen samples were mixed with 5 mL physiological saline and centrifuged at 8000× *g* for 10 min, and the supernatant was used for subsequent assay. The absorbance was recorded at 405 nm (H_2_O_2_ reacts with ammonium molybdate to form a faint yellow complex which has a characteristic absorption peak at this wavelength) and used for the calculation of H_2_O_2_ content, which was expressed as mmol kg^−1^ based on fresh weight.

### 2.7. Suberin Polyphenolic (SPP) and Lignin Deposition at Wounds

The wounded surface of the tested tubers was cut into thin vertical slices with a blade and washed with distilled water to remove starch particles. The SPP deposition was visualized according to the method as previously described [18] using microscopy (BX53, Olympus, Tokyo, Japan). The slices were immediately immersed in 1% (*w*/*v*) phloroglucinol solution for 2 h and stained on a glass slide with a few drops of concentrated hydrochloric acid according to the method as previously described [19]. After 5 min, the images of red-stained deposited lignin were captured using microscopy (BX53, Olympus, Japan). IS Capture software was used to measure the thickness of the cell layer in SPP and lignin.

### 2.8. Statistical Analysis

All experiments were repeated three times. The calculation of average value and standard deviation (±SD) of the data was carried out using SigmaPlot 12.0, followed by a Student’s *t* test. *p* < 0.05 or < 0.01 were of significance or extreme significance, respectively. 

## 3. Results

### 3.1. Acquisition and Verification of StMSD Interference-Expression Plantlets and Tubers

**Figure 1:**
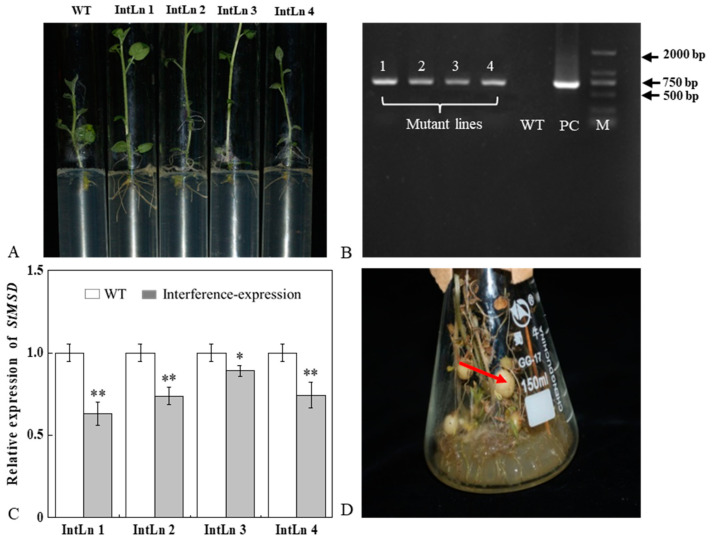
Acquisition and verification of *StMSD* interference-expression potato plantlets and tubers: (**A**) rooting screening of *StMSD*-interference plantlets; (**B**) PCR identification of genomic DNA from Kana-resistant potato plantlets, in which 1–4 are the obtained interference-expression plantlets, WT is the wild type plantlets, PC is the positive control, M is a 2 Kb ladder Marker; (**C**) quantitative RT-PCR analysis of *StMSD* in the interference-expression plantlets; (**D**) induction of interference-expression potato tubers (the red arrow shows the tubers). Bars indicate standard deviation (±SD). Asterisks represent significant differences (* *p* < 0.05; ** *p* < 0.01).

Four *StMSD*-interference plantlets were successfully obtained by root screening and named as IntLn 1, IntLn 2, IntLn 3, and IntLn 4 (Figure 1A). About 650 bp DNA fragments of *NPT II* gene were amplified and detected in the four lines and recombinant plasmid, and no bands were observed in untransformed plantlets (Figure 1B). The *StMSD* expressions in the four lines were lower than those in the wild type by 36.8%, 26.3%, 10.8%, and 25.6%, and the IntLn 1 line showed the most downregulation (Figure 1C). The *StMSD*-interference microtubers were obtained by further culturing the four lines (Figure 1D).

### 3.2. The Interference Expression of StMSD Affected the Expression of StSODs during Healing

**Figure 2:**
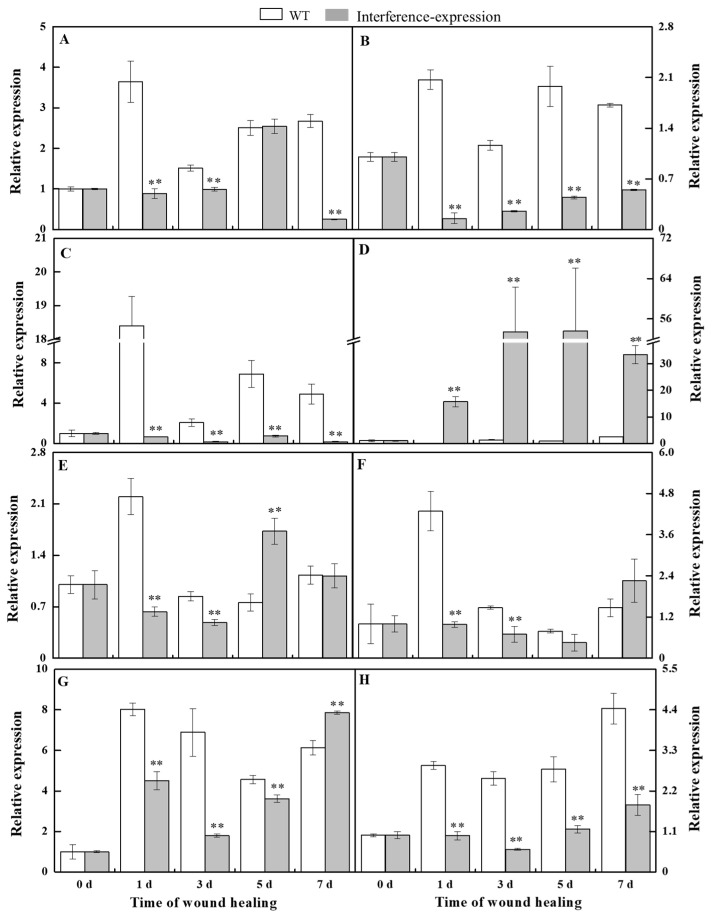
The interference expression of *StMSD* downregulated the gene expression of most *StSODs* genes during healing ((**A**): *StCSD1*; (**B**): *StCSD2*; (**C**): *StCSD3*; (**D**): *StCCS*; (**E**): *StFSD1*; (**F**): *StFSD2*; (**G**): *StFSD3*; (**H**): *StMSD*). The potato elongation factor 1-alpha 1 (*efla*) was used as an internal control to normalize the data. Bars indicate standard deviation (±SD). Asterisks represent significant differences (* *p* < 0.05; ** *p* < 0.01). Each column represents the mean of three replicates. The healing was carried out at ambient temperature (20–25 °C; RH 80–90%) in the dark. The four RNAi lines were used in the experiment.

The relative level of *StSODs* was examined to investigate whether the interference expression of *StMSD* affected the expression of its family genes during healing (Figure 2). The *StCSD1* in the *StMSD*-interference tubers showed a decreased expression level except at 5 d, which was 9.3% lower of the wild-type tubers at 7 d. The *StCSD2* and *StCSD3* also had lower expression levels, which were 7.4% and 3.5% lower than the wild-type at 1 d, respectively. On the contrary, the *StCCS* (*copper chaperone superoxide dismutase*) exhibited an increased expression level in the *StMSD*-interference tubers and was 264-fold of the wild-type at 1 d. Except for 5 d and 7 d, the *StFSD1* expression was lower than that in the wild-type tubers, which was 28.7% of wild-type at 1 d. The *StFSD2* and *StFSD3* expression of the interference-expression tubers also showed a lower level except at 7 d, which were 22.8% and 26% lower of the wild-type at 1 d and 3 d. The expression of *StMSD* had a clear decrease and was 24.3% lower than the wild-type at 3 d. The above results suggest that most *StSOD* expressions were downregulated in *StMSD*-interference tubers during healing, notably *StCSD2* and *StCSD3*. 

### 3.3. The Interference Expression of StMSD Inhibited the Disproportionation of O_2_^•−^ to H_2_O_2_ in Tubers during Healing

**Figure 3:**
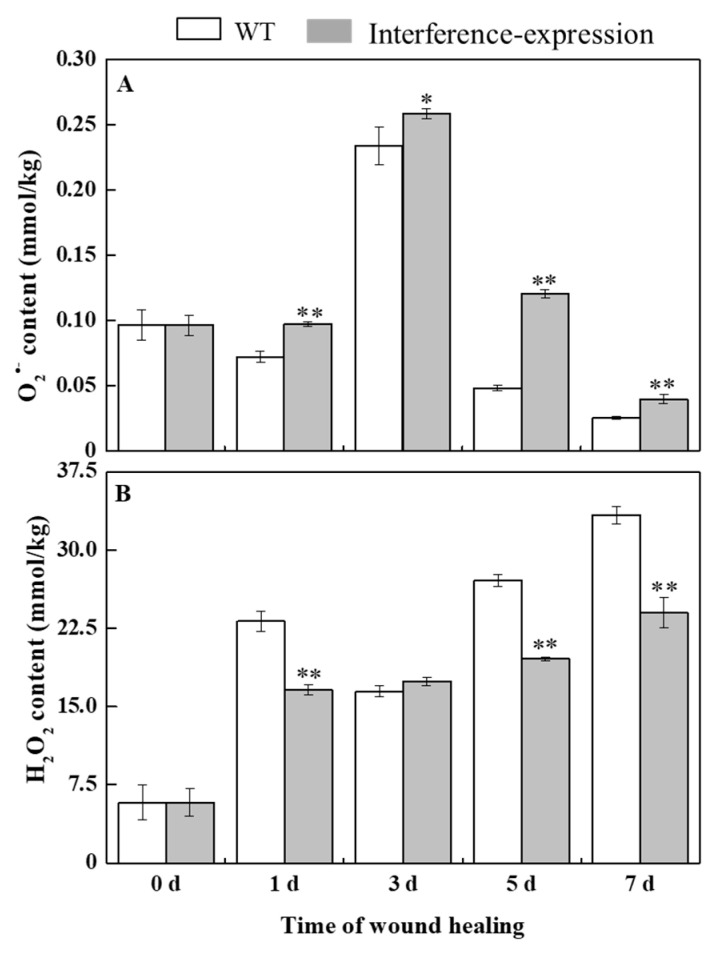
The interference expression of *StMSD* inhibited the disproportionation of O_2_^•^^−^ (**A**) to H_2_O_2_ (**B**) in tubers during healing. Bars indicate standard deviation (± SD). Asterisks represent significant differences (* *p* < 0.05; ** *p* < 0.01). Each column represents the mean of three replicates. The healing was carried out at ambient temperature (20–25 °C; RH 80–90%) in the dark. The four RNAi lines were used in the experiment.

O_2_^•−^ can be rapidly dismutated into H_2_O_2_ by SOD [6]. During healing, O_2_^•−^ content in *StMSD*-interference and wild type tubers showed a single peak at 3 d. The O_2_^•−^ content exhibited an obvious increase in the interference-expression tubers and was 2.48 times of the wild type at 5 d (Figure 3A). H_2_O_2_ content in the *StMSD*-interference and wild-type tubers increased gradually over time. Except for 3 d, H_2_O_2_ content showed a clear decrease in the interference-expression tubers, which was 71.8% of the wild type at 7 d (Figure 3B). The above results suggest that interference expression of *StMSD* inhibited the disproportionation of O_2_^•−^ to H_2_O_2_ in tubers during healing.

### 3.4. The Interference Expression of StMSD Downregulated the Gene Expression of StPAL and StC4H in Tubers during Healing

**Figure 4:**
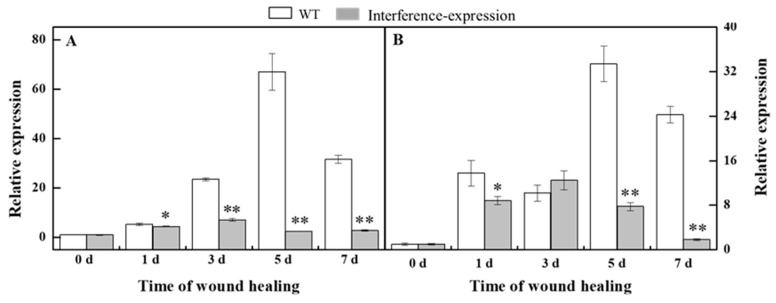
The interference expression of *StMSD* downregulated the gene expression of *StPAL* (**A**) and *StC4H* (**B**) in tubers during healing. The potato elongation factor 1-alpha 1 (*efla)* was used as an internal control to normalize the data. Bars indicate standard deviation (±SD). Asterisks represent significant differences (* *p* < 0.05; ** *p* < 0.01). Each column represents the mean of three replicates. The healing was carried out at ambient temperature (20–25 °C; RH 80–90%) in the dark. The four RNAi lines were used in the experiment.

Phenylpropanoid metabolism is essential for healing of tubers, and H_2_O_2_ is considered as a signal molecule that regulates phenylpropanoid metabolism [10,20]. During healing, the expression of *StPAL* in *StMSD*-interference was downregulated, which was 3.7% of the wild type at 5 d (Figure 4A). Similarly, the *StC4H* expression also showed a noticeable decrease in the interference-expression tubers except at 3 d, which was 7.5% of the wild type at 7 d (Figure 4B). The above results show that the interference expression of *StMSD* markedly downregulated the *StPAL* and *StC4H* expression in tubers during healing.

### 3.5. The Interference Expression of StMSD Resulted in Less Deposition of SPP and Lignin at Tubers Wounds during Healing

**Figure 5:**
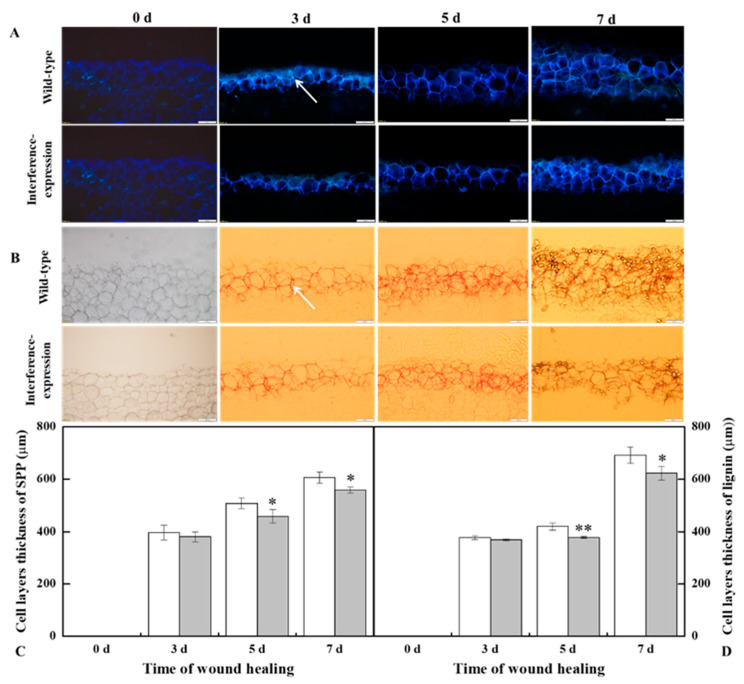
The interference expression of *StMSD* resulted in the less deposition of SPP (**A**,**C**) and lignin (**B**,**D**) at wounds of tubers during healing (arrows point at the SPP and lignin). Magnification = 10×, scale bar = 200 μm. Bars indicate standard deviation (±SD). Asterisks represent significant differences (* *p* < 0.05; ** *p* < 0.01). Each column represents the mean of three replicates. The healing was carried out at ambient temperature (20–25 °C; RH 80–90%) in the dark. The RNAi line IntLn-1 was used in the experiment.

The SPP and lignin deposition at tuber wounds reflects the healing ability of the potato tubers. During healing, interference expression of *StMSD* obviously inhibited the SPP and lignin deposition at wounds. The deposition amount in the interference-expression tubers was notably less than that in the wild-type tubers (Figure 5A,B). Similarly, interference expression of *StMSD* also decreased the thickness of SPP and lignified cell layers at wounds, which were 31.7% and 9.9% lower than the wild type tubers at 7 d (Figure 5C,D). Thus, the interference expression of *StMSD* inhibited the SPP and lignin deposition at tuber wounds.

## 4. Discussion

This study has reported that the change in a single SOD gene expression was closely correlated with the concentration change in O_2_^•−^ and H_2_O_2_, coupled with the change in other SOD family gene expressions [21]. The overexpression of *NtMSD* reduced the O_2_^•−^ concentration and inhibited the *NtCSD* expression [22]. The interference expression of *AtFSD1* and *AtFSD2* upregulated the *AtCSD2* expression in *Arabidopsis* [23]. In this study, the interference expression of *StMSD* downregulated the expression levels of its own and most *StSOD* family members to a certain extent and inhibited the disproportionation of O_2_^•−^ to H_2_O_2_ during healing in tubers (Figure 2 and Figure 3), which was similar to the result that the downregulated expression of *AtMSD1* and *AtMSD2* led to a decreased disproportionation ability of O_2_^•−^ in *Arabidopsis* leaves and roots, respectively [24,25]. It has been reported that CCS is required for the activation of CSD with the participation of O_2_^•−^ [26]. However, in this study, the upregulation of *StCCS* expression and accumulation of O_2_^•−^ level did not increase the expression of *StCSDs*, which was likely due to multiple factors involved in the regulation of *StCSDs* gene [27]. In addition, the interference-expression tubers also showed a decline in *StFSDs* expression levels, indicating that *StMSD* may interfere with the expression of other *StSODs* by an unknown mechanism.

H_2_O_2_ is considered as a significant signal molecule that activates phenylpropanoid metabolism in the tuber healing process [10]. The increased H_2_O_2_ content caused by overexpressing *PaSOD* in potato plants upregulated the *PAL*, *C4H*, and *4CL* expression in the phenylpropanoid metabolism [11]. Phenylpropanoid metabolism has been classified as an important contributor to providing not only phenolic acids for SPP polymerization but also corresponding lignin monomers for lignin synthesis at wounds [20,28]. PAL and C4H are two key enzymes of the phenylpropanoid metabolism; the former catalyzes L-phenylalanine to *trans*-cinnamic acid, and the latter converts the *trans*-cinnamic acid into *p*-coumaric acid, becoming the basis for the formation of phenolic acids and lignin monomers through a series of enzymes [29]. A previous study revealed that *StPAL1* and *StC4H* were actively involved in the formation of phenolic substances required for healing [30]. In this study, the downregulated expression of *StPAL* and *StC4H* was determined in the *StMSD* interference-expression tubers (Figure 4), indicating that *StMSD* gene may participate in the activation of phenylpropanoid metabolism by regulating H_2_O_2_ signaling during healing. As previously reported, H_2_O_2_ could assume a pivotal role in the induction of *PAL* expression in tomatoes as a direct signal [31]. Hence, it is presumed that the interference expression of *StMSD* may suppress phenylpropanoid metabolism by reducing H_2_O_2_ production. Although phenolic acids and lignin monomers, as the metabolites of phenylpropanoid metabolism, contribute to the formation of SPP and lignin at tuber wounds, there are still some phenolic acids and flavonoids that participate in scavenging of the free radical, which keeps the ROS homeostasis of wounds during healing [32]. 

SPP and lignin are essential constituents in tuber wounds, as they can provide a solid barrier for wounds [33,34]. The formation of SPP is initiated by the oxidative polymerization of phenolic acid monomers with an H_2_O_2_-mediated process [35]. Lignin consists of sinapyl alcohol, coniferyl alcohol, and *p*-coumaryl alcohol via oxidative crosslinking of H_2_O_2_ and peroxidase [36]. The formation of SPP and lignin provides a strong protection enabling tubers to resist pathogenic infections and to reduce water transpiration [33]. In this study, the less deposition of SPP and lignin at wounding sites was observed in the *StMSD* interference-expression tubers (Figure 5). This is because, on one hand, decreased H_2_O_2_ content led to the inhibition of the phenylpropanoid metabolism, which declined the substrates of SPP and lignin [37,38]. On the other hand, the reduction in H_2_O_2_ accumulation inhibited the oxidative crosslinking of corresponding monomers [33].

## 5. Conclusions

In this study, interference expression of *StMSD* markedly downregulated the expression of most *StSOD* family genes in tubers, notably *StCSD2* and *StCSD3*. The interference expression of *StMSD* also caused a remarkable increase in O_2_^•^^−^ content but decreased H_2_O_2_ accumulation. In addition, the interference expression of *StMSD* downregulated the *StPAL* and *StC4H* expression, thus leading to less SPP and lignin deposition at potato wounds during healing. These results reveal that the *StMSD* gene plays an essential role in the healing of potato tubers.

## Data Availability

All of the data is contained within the article and the supplementary materials.

## Supplementary Materials

The following supporting information can be downloaded at: https://www.mdpi.com/article/10.3390/antiox11101901/s1, Table S1. ‘Primer sequences of *StSODs*, *StPAL*, *StC4H* and housekeeping gene’.

## Abbreviations

SOD	Superoxide dismutase
SPP	Suberin polyphenolic
PAL	Phenylalanine ammonia lyase
C4H	Cinnamate 4-hydroxylase
NOX	NADPH oxidase
CSD	Cu/Zn superoxide dismutase
FSD	Fe superoxide dismutase
MSD	Mn superoxide dismutase
MS	Murashige and Skoog
PCR	Polymerase chain reaction
NCBI	National Center for Biotechnology Information
CCS	Copper chaperone superoxide dismutase

## References

[1] Dastmalchi K., Wang I., Stark R.E. (2016). Potato wound-healing tissues: A rich source of natural antioxidant molecules with potential for food preservation. Food Chem..

[2] Marhava P., Hoermayer L., Yoshida S., Marhavý P., Benková E., Friml J. (2019). Re-activation of stem cell pathways for pattern restoration in plant wound healing. Cell.

[3] Krknen A., Kuchitsu K. (2015). Reactive oxygen species in cell wall metabolism and development in plants. Phytochemistry.

[4] Ma L., Jiang H., Ren Y.Y., Yang J.W., Han Y., Si H.J., Prusky D., Bi Y., Wang Y. (2022). Overexpression of *StCDPK23* promotes wound healing of potato tubers by regulating *StRbohs*. Plant Physiol. Biochem..

[5] Jiang H., Wang Y., Bi Y. (2019). The process, mechanism and influence factors on wound healing of potato tubers. Acta Hortic. Sin..

[6] Ren Y.Y., Jiang H., Ma L., Li Y.C., Prusky D., Bi Y. (2021). Identification of potato SOD gene family and its response in damaged tubers. J. Agri. Biotechnol..

[7] Perry J., Shin D.S., Getzoff E.D., Tainer J.A. (2010). The structural biochemistry of the superoxide dismutase. Biochim. Biophys. Acta.

[8] Mahajan N.S., Mishra M., Tamhane V.A., Gupta V.S., Giri A.P. (2014). Stress inducible proteomic changes in *Capsicum annuum* leaves. Plant Physiol. Biochem..

[9] Perl-Treves P., Galun E. (1991). The tomato Cu, Zn superoxide dismutase genes are developmentally regulated and respond to light and stress. Plant Mol. Biol..

[10] Jacobo-Velázouez D.A., González-Agüero M., Cisneros-Zevallos L. (2015). Cross-talk between signaling pathways: The link between plant secondary metabolite production and wounding stress response. Sci. Rep..

[11] Shafi A., Pal A.K., Sharma V., Kalia S., Kumar S., Ahuja P.S., Singh A.K. (2017). Transgenic potato plants overexpressing SOD and APX exhibit enhanced lignification and starch biosynthesis with improved salt stress tolerance. Plant Mol. Biol. Rep..

[12] Karpinska B., Karlsson M., Schinkel H., Streller S., Süss K.H., Melzer M., Wingsle G.A. (2001). Novel superoxide dismutase with a high isoelectric point in higher plants. Expression, regulation and protein localization. Plant Physiol..

[13] Ogawa K., Kanematsu S., Asada K. (1997). Generation of superoxide anion and localization of CuZn-superoxide dismutase in the vascular tissue of spinach hypocotyls: Their association with lignification. Plant Cell Physiol..

[14] Wang X.Q., Shen X., He Y.M., Ren T.N., Wu W.T., Xi T. (2011). An optimized freeze-thaw method for transformation of *Agrobacterium tumefaciens* EHA 105 and LBA4404. Pharm. Biotechnol..

[15] Si H.J., Xie C.H., Liu J. (2003). An efficient protocol for *Agrobacterium*-mediated transformation of microtuber and the introduction of antisense class I patatin gene into potato. Acta Agron. Sin..

[16] Ma L., Jiang H., Bi Y., Li Y.C., Yang J.W., Si H.J., Ren Y.Y., Prusky D. (2021). The interaction between *StCDPK14* and *StRbohB* contributes to BTH-induced wound healing of potato tubers by regulating ROS generation. Front. Plant Sci..

[17] Schmittgen T.D., Livak K.J. (2008). Analyzing real-time PCR data by the comparative C (T) method. Nat. Protoc..

[18] Jiang H., Wang B., Ma L., Zheng X.Y., Gong D., Xue H.L., Bi Y., Wang Y., Zhang Z., Prusky D. (2019). Benzo-(1, 2, 3)-thiadiazole-7-carbothioic acid s-methyl ester (BTH) promotes tuber wound healing of potato by elevation of phenylpropanoid metabolism. Postharvest Biol. Technol..

[19] Oirschot Q.E.A.V., Rees D., Aked J., Kihurani A. (2006). Sweet potato cultivars differ in efficiency of wound healing. Postharvest Biol. Technol..

[20] Yang L.W., Bernards M.A. (2007). Metabolite profiling of potato (*Solanum tuberosum* L.) tubers during wound-induced suberization. Metabolomics..

[21] Foyer C.H., Noctor G. (2003). Redox sensing and signaling associated with reactive oxygen in chloroplasts, peroxisomes and mitochondria. Physiol. Plantarum..

[22] Slooten L., Capiau K., Camp W.V., Montagu M.V., Inzé D. (1995). Factors affecting the enhancement of oxidative stress tolerance in transgenic tobacco overexpressing manganese superoxide dismutase in the chloroplasts. Plant Physiol..

[23] Myouga F., Hosoda C., Umezawa T., Iizumi H., Kuromori T. (2008). A heterocomplex of iron superoxide dismutases defends chloroplast nucleoids against oxidative stress and is essential for chloroplast development in *Arabidopsis*. Plant Cell..

[24] Morgan M.J. (2008). Decrease in manganese superoxide dismutase leads to reduced root growth and affects tricarboxylic acid cycle flux and mitochondrial redox homeostasis. Plant Physiol..

[25] Chen H.Z., Lee J.S., Lee J.M., Han M., Emoner A., Lee J., Jia X.T., Lee Y. (2021). MSD2, an apoplastic Mn-SOD, contributes to root skotomorphogenic growth by modulating ROS distribution in *Arabidopsis*. Plant Sci..

[26] Brown N.M., Torres A.S., Doan P.E., O’Halloran T.V. (2004). Oxygen and the copper chaperone CCS regulate posttranslational activation of Cu, Zn superoxide dismutase. Proc. Nat. Acad. Sci. USA.

[27] Rae T.D., Torres A.S., O’Halloran T.V. (2001). Mechanism of Cu, Zn-superoxide dismutase activation by the human metallochaperone hCCS. J. Biol. Chem..

[28] Boudet M.A. (2000). Lignin and lignification: Selected issues. Plant Physiol. Biochem..

[29] Yu X.Y., Bi Y., Yan L., Liu X., Wang Y., Shen K.P., Li Y.C. (2016). Activation of phenylpropanoid pathway and PR of potato tuber against *Fusarium sulphureum* by fungal elicitor from *Trichothecium roseum*. World J. Microbiol. Biotechnol..

[30] Woolfson K.N., Haggitt M.L., Zhang Y.N., Kachura A., Bjelica A., Rincon M.A.R. (2018). Differential induction of polar and non-polar metabolism during wound-induced suberization in potato (*Solanum tuberosum* L.) tubers. Plant J..

[31] Gayoso C., Pomar F., Novo-Uzal E., Merino F., De Ilárduya Ó.M. (2010). The *Ve*-mediated resistance response of the tomato to *Verticillium dahliae* involves H_2_O_2_, peroxidase and lignin and drives *PAL* gene expression. BMC Plant Biol..

[32] Xie P.D., Yang Y.Y., Gong D., Yu L.R., Han Y., Zong Y.Y., Li Y.C., Prusky D., Bi Y. (2022). Chitooligosaccharide maintained cell membrane integrity by regulating reactive oxygen species homeostasis at wounds of potato tubers during healing. Antioxidants.

[33] Lulai E.C., Campbell L.G., Fugate K.K., McCue K.F. (2016). Biological differences that distinguish the two major stages of wound healing in potato tubers. Plant Signal. Behav..

[34] Dora D.S.C., Alviano Moreno D.S., Alviano C.S., Antonio Jorge R.D.S. (2022). Extension of *Solanaceae* food crops shelf life by the use of elicitors and sustainable practices during postharvest phase. Food Biopro. Technol..

[35] Razem F.A., Bernards M.A. (2002). Hydrogen peroxide is required for poly (phenolic) domain formation during wound-induced suberization. J. Agric. Food Chem..

[36] Eisenstadt M.A., Bogolitsyn K.G. (2010). Peroxidase oxidation of lignin and its model compounds. Russ. J. Bioorganic Chem..

[37] Ramamurthy M.S., Ussuf K.K., Nair P.M., Thomas P. (2000). Lignin biosynthesis during wound healing of potato tubers in response to gamma irradiation. Postharvest Biol. Technol..

[38] Lin J.S., Lin C.C., Lin H.H., Chen Y.C., Jeng S.T. (2012). MicroR828 regulates lignin and H_2_O_2_ accumulation in sweet potato on wounding. New Phytol..

