# Supplementary material for: Interference Expression of StMSD Inhibited the Deposition of Suberin and Lignin at Wounds of Potato Tubers by Reducing the Production of H2O2
Source: Antioxidants (Basel). 2022 Sep 25;11(10):1901. doi: 10.3390/antiox11101901 (PMC9598499; doi:10.3390/antiox11101901)
Supplement: Supplementary file 1 [file antioxidants-11-01901-s001.zip › antioxidants-1883544-supplementary.pdf]

Table S1. Primer sequences of *StSODs*, *StPAL*, *StC4H* and housekeeping gene.

Primer name	NCBI Gene ID	Soltu Gene ID	Forward primer (5'-3')	Reverse primer (5'-3')
StCSD1	NM_001288080.1	Soltu.DM.01G022650	CGACCAGCAGATTCCTCTC	ATCCTTCCGCCAGCATTTC
StCSD2	XM_006349513.2	Soltu.DM.03G010200	GGTACCCATGTGAGAGGAC	ATGTTGCCTAAATCACCCG
StCSD3	XM_015309446.1	Soltu.DM.11G020830	TCAATCTTCACCACAACCAG	AACAGTAAGGGGTTTGGG
StCCS	XM_006363638.2	Soltu.DM.08G026370	TCGCAGTTTGAACGTCCATC	TTGGTGATTGGAAGAAGG
StFSD1	XM_006350307.2	Soltu.DM.02G001300	TCCACTTCAAGTTCGTCGGT	CCACGATGATGGTCTCCCC
StFSD2	XM_006358116.2	Soltu.DM.03G013800	CTCCATTTCTTCCTTCCCCAG	GTGATGCTTCCCCCAGTGA
StFSD3	XM_006357250.2	Soltu.DM.06G012180	ATTCAACAATGCTGCTCAG	GTATCATAGGAACCAAAGT
StMSD	XM_006358693.2	Soltu.DM.06G011380	TTCTCTTGGCTGGGCTATCG	AAGGGTCCTGATTTGCAG
Stef1-a	AB061263	Soltu.DM.06G005620	ATTGATGCCCCTGGTCACAG	CATGTTCACGGGTCTGACC
StPAL	X63103.1	Soltu.DM.03G011480	CAAACTTGACGCTGATGAAG	ACAGGACAATTGATGCCAT
StC4H	DQ341174.1	Soltu.DM.06G032860	ACCAAGAGCATGGACAGCA	ATCCTCGTTGATCTCTCCC
